# Supplementary material for: Decoding biomolecular condensate dynamics: an energy landscape approach
Source: PLoS Comput Biol. 2025 Feb 10;21(2):e1012826. doi: 10.1371/journal.pcbi.1012826 (PMC11841893; doi:10.1371/journal.pcbi.1012826)
Supplement: Attachment — Submitted filename: response2rev.pdf [file pcbi.1012826.s002.pdf]

Reviewer 1

Reviewer's Comment*: The authors provide a minimal model to characterize the equilibrium and non-equilibrium behaviors of 'protein' condensates. Instead of considering the exact identity of the amino acids for instance they consider the relative binding strength of interaction sites and define these binding interactions in a continuous manner – yielding an 'energy landscape' of interactions that can be arranged in a periodic fashion to decode the role on thermodynamic and transport properties of condensates. The manuscript is very well-written, the analyses are rigorous and the paper features quite high-quality figures/images. There are several coarse-grained models and sticker-spacer type models that have been developed and used to interrogate similar questions. Where this approach is different is in the generality of how it can be used to investigate how properties of condensates are encoded by their sequences. The paper is already at a high standard, but there are some areas where the narrative can be improved/where details are needed to fully appreciate that value of this contribution/reproduce the work.*

Response: We greatly appreciate the reviewer's positive feedback on the novelty and significance of our work. We are also thankful for the thoughtful and constructive comments, which have prompted us to revise the manuscript thoroughly, resulting in substantial improvements. We have carefully addressed all the concerns raised by the reviewer, as reflected in the updated manuscript and supplementary information. Furthermore, we have made additional enhancements to reinforce our arguments and improve the overall quality and rigor of the paper.

Reviewer's Comment: Q 1*. The authors should state upfront clearly what they mean by alphabet-free. This language is somewhat abstract.*

Response*:* We thank the reviewer for highlighting this point. In the manuscript, we explicitly clarify the term 'alphabet-free', which signifies that the model does not rely on the amino acid sequence. Instead, it utilizes a continuous energy scale to uncover the fundamental features of the energy landscape that govern the viscoelastic dynamics of condensates.

Reviewer's Comment : Q 2*. However, use of an 'alphabet-free' model in probing these properties is appealing, as it allows for generality but is still detailed enough to capture effects of patterning and composition. However, the constant use of the term "sticker-spacer" throughout the paper defeats the purpose of an 'alphabet-free' approach / seems contradictory. The fact is it is almost impossible to delineate protein residues as pure stickers or spacers, which is exactly why their model is a good way of addressing this. But then in calling things stickers and spacers the authors are making assignments that are not needed. It also causes one to think of this problem in a binary way, which is not what they are doing or intend to do. It would be beneficial to get rid of this language and state clearly why their model is a better way at approaching this problem.*

Response: We thank the reviewer for this thoughtful comment. We agree that the frequent use of the terms "sticker" and "spacer" could unintentionally obscure the 'alphabet-free' nature of our model and lead to an overly binary interpretation of the system. To address this, we reduced the use of this terminology throughout the manuscript. However, we kept minimal references to these terms where they do provide helpful description the extremes of interactions such as highly attractive or non-attractive beads within the context of our energy scale. This approach allows us to clarify the conceptual framework of the model while maintaining the generality and non-binary perspective. Additionally, we revised the text to more clearly articulate why

our model is particularly well-suited for addressing the complexity of patterning and composition beyond simplistic categorizations.

***Reviewer's Comment: Q**3. The abstract includes a lot of field-specific language that would make the work less accessible to abroad audience. A key message is that these interactions exist on a continuum and that the context matters. Making it clear what this model offers, how it differs from other coarse-grained approaches (of which there are many), and what is the value in approaching the problem in the way they do would is needed. What is the benefit of this approach versus use assigning a hydropathy score to residues?*

Response: We thank the reviewer for this point and agree that a clean comparison of our model from the widely used models based on the Hydropathy Scale is warranted. In short, the ability of an energy landscape model to continuously vary the energy scale of the interaction of coarse-grained beads, as opposed to having explicit sequence with multiple potentials, allows us to dissect the key features that contribute to mesoscopic material properties such as viscosity and viscoelasticity.

***Reviewer's Comment: Q**4. The introduction stresses connections to energy landscape theory but this does not pay off upon reading the paper. In the discussions connecting back to the idea that the underlying sequence encodes the thermodynamics and dynamics of condensates in a similar way in which it encodes behavior of single proteins would be powerful in driving their message.*

Response: This is an excellent suggestion. We have now add sentences that a) Connects the novel approach of this manuscript to the energy landscape theory used in pioneering studies of protein folding and binding which focuses on dynamics of single biomolecules! b) We also clearly state difference of the present energy landscape framework from the ones used in the field of protein folding.

***Reviewer's comment: Q**5. How do they envision others using this approach? Is there a set prescription for mapping real proteins onto their energy landscape framework? Section 3.3 provides some insight, but this needs to be stated more clearly.*

Response: We thank the reviewers for raising this point. The unique advantage of our framework, compared to sequence-resolved models, lies in its ability to identify general energetic patterning that underpins the mesoscopic viscoelastic dynamics observed in experiments. With the energetic pattern in hand, one can map it onto various sequences using frameworks such as HPS or even atomistic pairwise residue energy scales. The results presented in this manuscript suggest that for a given energetic pattern or landscape, multiple sequence mappings are possible! In future studies, it would be intriguing to quantitatively explore the degree of this degeneracy for specific patterns. We have now incorporated a discussion of these applications and potential future developments into the manuscript.

***Reviewer's comment: Q**6. In figure 3C, explain the trend between the blue curves. Why is it non-monotonic compared to the periodicity?*

Response: Thank you for highlighting this point. This non-monotonic trend among P_n, the blue curves with increasing n, arises from the combined effects of system dynamics and spatial organization. As the sticker period n increases, the systems exhibit varying levels of arrest. For P_{10}, the sticker bands are less stable and more dynamic, causing the system to behave similarly to randomly arranged sequence chains at longer times. In contrast, P_{25} initially shows an increase in behavior akin to random chains but later deviates and decreases, following a 1/5-power law. The behavior of P_{100} is interesting, as it shows two distinct scaling regimes. Due to its long spacer regions, P_{100} initially behaves like random chains for shorter root-mean-

squared distances. However, as the spacer dynamics exceed the spacer mesh size, the system becomes increasingly arrested, causing the P_{100} curve to surpass those of lower P_n values. This anomaly highlights the interplay between sticker periodicity and spacer fluctuations, which significantly influences long-time behavior. We have ensured that this trend is not due to insufficient sampling. The simulations were conducted over sufficiently long timescales to capture the dynamics of all P_n systems, confirming that the trends in Fig. 3C are robust and accurately reflect the system's underlying dynamics. We have incorporated it in the main text marked in blue.

Minor remarks:

Reviewer's comment: Q*7. Figures are not referenced in the order they appear. E.g., Fig. 4 in section 2 before mentioning Fig 3.*

Response: Thank you for pointing this out. To clarify, Fig. 4 is referenced earlier in Section 2 to provide the chain schematic, which is essential for understanding the system setup before discussing the results presented in Fig. 3. However, we recognize the importance of maintaining the sequential referencing of figures. To address this, we have inserted a statement to introduce the context of Fig. 3 before referring to Fig. 4, ensuring a logical flow and compliance with figure referencing conventions.

Reviewer's comment: Q*8. The chain length of 200 does not mimic condensate-forming proteins. (Line 100). This is mimicking LCDs of condensate-forming proteins.*

Response: Thank you for highlighting this point. We agree that the original phrasing could lead to confusion. To clarify, we have revised the statement to reflect this clarification.

Reviewer's comment: Q*9. 'mere shuffling' what does this mean? What is the procedure for generating the randomized sequences.*

Response: Thank you for pointing out the need for clarification. The term 'mere shuffling' refers to the process of randomizing the spatial arrangement of sticker and spacer motifs in the periodic sequence. Specifically, to generate the randomized sequences, we took the chain with a periodic arrangement (e.g., P_{100}) and shuffled the sequence positions by randomly generating a new sequence array using MATLAB's randi function (pseudorandom number generator). This procedure ensures that the overall composition of motifs remains unchanged, but their spatial order is randomized. We have revised the manuscript to replace "mere shuffling" with a more precise description of the randomization procedure to avoid ambiguity and provide clarity for the readers.

Reviewer's comment: Q*10. Some places use 'sticker spacers' what is this? This phrasing is confusing/dilutes their message.*

Response: Thank you for pointing out the potential ambiguity in the phrase 'sticker spacers'. We agree that this phrasing could be confusing and might dilute the clarity of the message. To address this, we have revised the manuscript to consistently use 'sequences composed of stickers and spacers', which more clearly conveys the intended meaning.

Reviewer 2

Reviewer's Comment: *In this work, the authors present Alphabet-Free Energy Landscape framework of Stickers and Spacers (AFELAS) in which protein sequence is defined by*

specifying the relative "sticker" affinity of residues with respect to baseline "spacer" affinity. This study investigates how protein sequence patterns influence the material properties of biomolecular condensates through a so-called "energy landscape" framework. By using molecular dynamics simulations of a simple spring-bead polymer model, the authors show that periodic sticker patterns promote the formation of elasticity-dominated, stable condensates, while random patterns lead to viscosity-dominated, less stable condensates. I LOVE this work. The authors thoroughly investigated the equilibrium and kinetic material properties with appropriate analysis methods. They compare their results with available experimental data (although there are very few, unfortunately). The structure of the manuscript is clear and concise. Their findings underscore the importance of sequence periodicity in determining condensate stability and fluidity, providing valuable insights into protein aggregation-related diseases and the design of synthetic biomaterials. I would strongly recommend my students to study this work as a role model. I just provide several comments below, which may help the authors improve the manuscript. The list is somewhat lengthy, but the comments are mostly editorial.

Response: We are thrilled to hear that the referee found our work interesting and appreciated the novel results. We are also grateful for numerous insightful comments which have led us to thoroughly revise the manuscript, making it significantly better. We have carefully addressed the concerns raised by the reviewer, which are reflected in the revised text and SI. We have incorporated additional changes to bolster our defense and strengthen our paper, believing that these additions enhance the overall quality and rigor of our work.

Reviewer's comment: Q*1. The authors call their approach the energy landscape framework, but I don't fully grasp the meaning. In the field of protein folding, the energy landscape is a well-defined object, where energy is represented as a function of reaction coordinates. In this work, the authors seem to use different sequence patterns as the "reaction coordinate," but then, what is a quantity corresponding to energy? If it is not precisely defined, I am not sure if you can call it the "energy landscape" framework.*

Response: We thank the reviewer for raising this important point. In this work, the term 'energy landscape' framework is used to describe the systematic approach of examining sequence-dependent interactions through a continuous energy scale. Here, different sequence patterns serve as the evolution of the sequence, and the corresponding energy is represented as the interaction energy, δE , between residues. Unlike traditional energy landscapes in protein folding, which focus on reaction coordinates for structural transitions, our framework is tailored to explore how sequence organization influences the mesoscopic thermodynamics and dynamics of condensates. This allows us to uncover general principles governing condensate behavior without requiring explicit structural resolution. We have clarified this distinction in the revised manuscript.

Reviewer's comment: Q*2. Since the main tool of this work is molecular dynamics simulations, I think that the authors should make sure if the simulated systems, particularly those for equilibrium properties, are fully converged. Can the authors add some discussion on this?*

Response: We thank the reviewer for this important comment. In this study, we have carefully ensured that the simulated systems, particularly those used to calculate equilibrium properties, are fully converged. To achieve this, we monitored key observables (e.g., total energy, radial distribution functions, and structure factors) over time to confirm that they reached steady-state values before data collection. Additionally, we conducted sufficiently long simulation runs (10^8 – 10^9 steps with step size Δt =0.005) a couple of times to take the average and verified that the results were independent of initial conditions by repeating simulations with different random seeds.

Reviewer's comment 3: *The authors fixed the chain lengths at Np=200, as exploring a distribution of chain lengths could improve the robustness of their findings. What is the main reason for choosing that? If there is any study published, please provide a reference.*

Response: We thank the reviewer for this insightful comment. In our study, we chose a fixed chain length of $N = 200$ beads based on both practical considerations and prior work. Specifically, this chain length aligns with the intrinsically disordered protein (IDP) length of the FUS protein, as studied in our previous work (Biswas and Potoyan, PRX Life 2024). Additionally, in Fig. 9, we varied chain lengths ($N = 25, 50, 100,$ and 200) for P_{25} sequences and demonstrated various trends in sticker cluster shape distributions and the absence of network structures for smaller chains (see also SI Fig. S4). This shows that the qualitative behavior of the system is robust across a range of chain lengths. By choosing a relatively long chain length ($N = 200$), we could explore the interplay between chain length and sticker periodicity. This allowed us to capture both fluidic and arrested dynamics, as well as the static behavior. For instance, shorter periodicity (P_{10}) results in a critical temperature that is approximately half that of chains with longer periodicity (P_{100}). Thus, the choice of $N = 200$ enables us to effectively analyze extremes of periodicity while maintaining a representative system size.

Reviewer's comment 4: *This model assumes constant interaction strengths for stickers, which might not capture context-dependent interactions such as pH or ionic strength variations in biological environments. Can the authors comment on how to incorporate such effects in this model?*

Response: In typical simulations, non-bonded interactions are fixed for individual beads. However, bead types could be swapped or adjusted to reflect changing environments, with interaction strengths modeled as functions of parameters like pH or ionic strength. Incorporating pH- or ion-sensitive scaling factors into the interaction matrix would allow dynamic adjustments during simulations. While these approaches are beyond the scope of the current study, which focuses on constant interaction strengths to highlight sequence periodicity and phase behavior, they represent a valuable direction for exploring environmental effects on phase separation.

Reviewer's comment 5: *The authors sometimes introduce symbols and abbreviations without their definitions. For example: 5-a. In Fig. 1, what is P(δE)? 5-b. In line 75, what is WCA? It is used again in line 81. 5-c. In Fig. S1, the authors used abbreviations like PDSSM and RDSSM, which are never defined anywhere in the main text or the SI.*

Response: We thank the reviewer for pointing out the typos and figure inconsistency.

5-a: $P(\delta E)$ is the probability distributions of the non bonded interaction energy. Incorporated in the figure caption.

5-b: WCA stands for Weeks-Chandler-Andersen potential. Incorporated in the main text.

5-c: We apologized for outseeing the abbreviations and replaced by periodically distributed sticker spacer motifs and randomly distributed sticker spacer motifs.

Reviewer's Comment 6: *In the caption of Fig. 1, the authors mention "motif IDs." How do the authors define the distinct motifs and their lengths?*

Response: In our model, unlike the simple case of two types of sticker and spacer beads, we define motifs based on varying sticker strengths. Each motif corresponds to a distinct bead with a unique interaction energy. To investigate the effect of periodicity, we use three distinct periodicities: 10, 25, and 100, which result in 6, 13, and 42 unique motif IDs, respectively (these numbers came from discretization of the $sin^2 2\pi\ Res_i/k$ as indicated in sec 2). These periodicities are chosen as factors of the total chain length (200 beads) to ensure evenly spaced arrangements along the chain. The chain length is fixed at 200 beads for all simulations, and the choice of periodicity ensures consistency in motif distribution while allowing us to explore how different sequence arrangements impact the system's behavior. Additional details about the definition and implementation of motifs are provided at the end of Section 2.

Reviewer's Question 7: *In the caption of Fig. 2, the authors mention "The interaction strengths between distinct stickers are illustrated in the energy landscape diagram." Which panel illustrates it?*

Response: We thank the reviewer for pointing out this typo. We have corrected it, marked in blue in the main text.

Reviewer's Question 8: *Can the authors explain or provide a rationale behind the statement (in line 109), "Intriguingly, regardless of the permutation of the periodically distributed chain, the phase diagram of the randomly arranged sequences manifests approximately the same critical temperatures…"?*

Response: The statement refers to the observation that the critical temperatures (T_c) for phase separation in the fully randomized arrangements (R_{10}, R_{25}, R_{100}) of sticker and spacer motifs are approximately the same, regardless of the periodicity or permutation of the original sequence. This behavior can be attributed to the loss of spatial correlation in the random sequences. In periodically distributed chains, stickers and spacers create regions of local structure that are difficult to disintegrate in small thermal fluctuations, which influence the critical temperature. However, in fully randomized arrangements, these spatial correlations are effectively erased. As a result, the critical temperature becomes dominated by the average sticker concentration and interaction energy rather than specific sequence motifs or arrangements. The approximate critical temperature ($T_c \sim 1.4\ \varepsilon$) reflects the fact that the phase behavior in these randomized systems is governed primarily by the overall fraction of stickers and their average interactions, rather than the details of the sequence arrangement, as illustrated in Fig3 (A).

Reviewer's Question 9: *I wonder why the authors use S(q) instead of g(r). Both contain the same information, but for the readers who are not familiar with the reciprocal space representation, g(r) may be easier to understand. Do the authors have specific reasons for using S(q)?*

Response: We appreciate the reviewer's observation. To address this point, we have included the radial distribution function *g(r)* as a function of *r* for comparison in the figure below and in the SI. As shown in the new figure, the *g(r)* analysis does not provide additional significant insights into the structural differences between P_i and R_i systems. The short-distance behavior is nearly identical for both, and *g(r)* effectively captures this aspect. However, *S(q)* proves more advantageous in highlighting the long-distance properties of the system, which are crucial for understanding sticker cluster formation. Specifically, the structure factor *S(q)* enables a more direct estimation of cluster sizes through its peak positions and intensity. Since the focus of this study is on longer lengthscale cluster formation and their spatial organization, we chose to emphasize *S(q)* over *g(r)*. For completeness, we now include the *g(r)* analysis as supplementary material to provide additional context for readers unfamiliar with reciprocal

space representations.

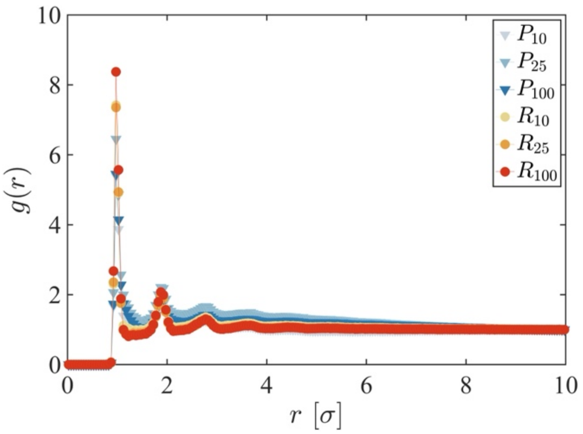

Reviewer's Question 10: *In Fig. 3C, the behaviors of the Pn systems require some explanation. The curves cross each other, and there is apparently no trend along increasing n. I am a little worried if this is due to insufficient sampling.*

Response: Thank you for highlighting this point. We have carefully investigated the behavior of the P_n systems, as shown in Fig. 3C, to address the observed crossing of curves and lack of a clear trend with increasing n. The behavior is indeed influenced by the dynamics and spatial organization of the systems. As the sticker period n increases, the systems exhibit varying degrees of arrest. For P_{10}, the sticker bands are less stable and more dynamic, resulting in behavior akin to random chains at long times. In contrast, P_{25} shows an initial increase similar to random chains but deviates and decreases over time, following a 1/5-power law. The behavior of P_{100} is particularly noteworthy. Due to its long spacer regions, P_{100} initially behaves similarly to random chains for shorter root-mean-squared distances. However, as the spacer dynamics surpass the spacer mesh size, the system becomes increasingly arrested, leading to a crossover where P_{100} curves exceed those of lower P_n values. This anomaly highlights the interplay between sticker periodicity and spacer fluctuations, which dominates the long-time behavior. We also confirm that this observation is not due to insufficient sampling. The simulations have been conducted over sufficiently long timescales to capture the dynamics of all P_n systems. This ensures that the trends observed in Fig. 3C are robust and accurately represent the underlying dynamics of the systems.

Reviewer's Question 11: *In section 3.3 or Fig. 5, the details of the experimental systems (such as protein names) should be provided.*

Response: We thank the reviewer for pointing out this issue. The protein sequence names are listed in the SI section D, also mentioned in the main text.

Reviewer's Question 12: *In Fig. 6B and 6D, why do we have "bumps" (significant deviations from the exponential decay) in the spacer distributions?*

Response: In Fig. 6 B&D, the bumps in the exponential decay represent two different scales of tangent correlation, which represents the variational persistence length entirely due to the interplay between the imposed angular potential and the sticker and spacer domain dimension. As they are similar in size, looking from a spacer particle perspective, it decays fast, however, once it hits the sticker

Reviewer's Question 13: *In Fig. 6F, it seems that the authors use the same curve for all three different systems. However, even when the three curves overlap, fluctuations may give some deviations. Hence, I recommend the authors use different colors for different curves.*

Response: We thank the reviewer for the recommendation. We have added the plot legends.

Reviewer's Question 14: *The caption for Fig 7 seems to be truncated.*

Response: We revisited the caption, which is indicated in blue in the main text.

Reviewer's Question 15: *In lines 290-291, what do the authors mean by "randomly distributed frustrated sticker-spacer condensed phase"?*

Response: The term "frustration" in the context of the 'randomly distributed frustrated sticker-spacer condensed phase' refers to a well-known phenomenon in condensed matter physics. Frustration arises when a system cannot simultaneously minimize all interaction energies due to geometric or spatial constraints. In our system, the random distribution of stickers (strongly interacting residues) and spacers (non-interacting residues) creates an inherent incompatibility that prevents the formation of an optimal, energetically favorable network of interactions. This frustration leads to a disordered structure within the condensed phase, significantly affecting the dynamical behavior, as evidenced by the deviations from Gaussian behavior in the self-van Hove function.

We also revise the main text in order to integrate the introduction of frustration in this context. These observations highlight the impact of spatial disorder and frustration on the dynamic properties of the condensed phase.

Reviewer's Question 16: *In lines 294-295, the authors claim that "randomly arranged stickers remain Gaussian in nature," but is it true? Fig. 7F seems to indicate that randomly arranged stickers deviate from the Gaussian behaviour as time progresses.*

Response: Thank you for bringing the typo to our attention. We have corrected the text indicated in blue.

Reviewer's Question 17: *In line 317, the authors state "However, Rgii degenerates as density increases." What do they mean by "degenerates"?*

Response: The term '*degenerates*' refers to the loss of equivalence among the three principal components of the gyration tensor, R_{xx}^g, R_{yy}^g, and R_{zz}^g. For spherical clusters, these components are equal, reflecting isotropic symmetry. These components become unequal as density increases, indicating a transition to an elongated cluster shape, where isotropy is lost. This behavior is consistent across different chain lengths, as described in Fig 8 (G).

Reviewer's Question 18: *In line 356, the authors claim that they continuously "evolve" sequence types. I believe that the authors mean "change." I strongly recommend replacing the word "evolve," as in the field of protein science, it is usually related to biological evolution*

Response: Thank you for pointing out the potential ambiguity in our use of the term 'evolve'. To avoid confusion, we have replaced the word '*evolve*' with '*systematically modify*' in the revised manuscript.

Minor Comments:

Reviewer's comment 1: *In line 15, is "membrane organless" a typo for "membraneless organelles"?*

Response: We thank the reviewer pointed out this typo. We corrected it and highlighted blue in the main text.

Reviewer's comment *2: In line 23, is "sequence-condensate thermodynamics" a typo for "sequence-dependent thermodynamics"?*

Response: We thank the reviewer pointed out this typo. We corrected it and highlighted blue in the main text.

Reviewer's comment *3. In line 33, can the authors provide some references for "evolutionary pressure optimizes the energy gap between unfolded and folded states at physiologic conditions to avoid deep kinetic traps thereby ensuring rapid and robust folding"?*

Response: We thank the reviewer pointed out this typo. We have added the references [22-24] mentioned in blue in the main text.

Reviewer's comment *4. In Figs. 2, 4 and S1, the texts are too small, and the images also seem squeezed. Can authors make it more readable?*

Response: We thank the reviewer pointed out these not-readable figure fonts. We have updated the figures and made them full-width figures in the main text.

Reviewer's comment *5. In line 201, 211, 330, is "micell-like" a typo for "micelle-like"?*

Response: We thank the reviewer pointed out this typo. We corrected it and highlighted blue in the main text.

Reviewer's comment *6. In lines 314-315, the phrase "where for chain lengths N p = 25, 50, 100 (Fig. 8 G)." is ungrammatical.*

Response: We thank the reviewer pointed out this typo. We corrected it and highlighted blue in the main text.

Reviewer's comment *7. In the first row of Fig. 9, the snapshots are overlapped with the panel labels and the texts. Can the authors rearrange them to show more clearly?*

Response: We thank the reviewer pointed out that figure labels and text overlapped. We corrected it.

Reviewer's comment *8. In line 329, "Whereas cos−1 Ξ delineates the transition between oblate and prolate shapes." is a fragmented clause.*

Response: We thank the reviewer pointed out this unclear statement. We corrected it and highlighted blue in the main text.

Reviewer's comment *9. In line 330, there is a comma before the colon. Is it a typo?*

Response: We thank the reviewer pointed out this typo. We corrected it and highlighted blue in the main text.

Reviewer's comment *10. In line 374, the hyphen in "The van-Hove analysis" should be removed.*

Response: We thank the reviewer pointed out this typo. We corrected it and highlighted blue in the main text.

Reviewer's comment *11. In line 378, is "micelles-like" a typo for "micelle-like"?*

Response: We thank the reviewer pointed out this typo. We corrected it and highlighted blue in the main text.

Reviewer's comment *12. In the SI, the first line repeats word "map" twice.*

Response: We thank the reviewer pointed out this typo. We corrected it and highlighted blue in the main text.
